# 748. The Changing Epidemiology of *Clostridioides difficile* Infection and the NAP1/027 Strain in Two Quebec Hospitals

**DOI:** 10.1093/ofid/ofab466.945

**Published:** 2021-12-04

**Authors:** Sandrine Couture, Charles Frenette, Rowin Alfaro, Lorne Schweitzer, Ian Schiller, Nancy Doherty, Rahul Nanda, Yves Longtin, Daniel Thirion, Vivian Loo

**Affiliations:** 1 McGill University Health Centre, Montreal, Quebec, Canada; 2 Jewish General Hospital, Montreal, Montreal, QC, Canada; 3 McGill University, Montreal, Quebec, Canada

## Abstract

**Background:**

In 2003, many hospitals in Québec, Canada experienced an increase in the incidence of healthcare-associated *C. difficile* infection (HA-CDI) associated with increased morbidity and mortality. This increase was associated with the dissemination of the NAP1/027 strain. The objective of this study was to describe the epidemiology of HA-CDI in two tertiary care hospitals based in Montréal from 2003 to 2019.

**Methods:**

Surveillance for HA-CDI was performed using standard definitions from 2003 to 2019 at the Montreal General Hospital (MGH) and Royal Victoria Hospital (RVH), in Montréal, Québec. *C. difficile* was isolated from stool specimens using standard methods. Pulsed field gel electrophoresis and ribotyping were performed to determine genotype. Antibiotic utilization and infection control interventions implemented over the same time period were reviewed.

**Results:**

A total of 4314 cases of CDAD were identified during the study period: 2295 at the RVH and 2019 at the MGH. The incidence decreased from 29.5 to 5.9 cases per 10,000 patient-days between 2003 and 2019 at the RVH and from 23.8 to 3.9 cases per 10,000 patient-days at the MGH. Of the 124 isolates available for genotyping in 2003, 112 were NAP1 (90.3%) compared to 5 out of 53 (9.4%) in 2019. Fluoroquinolone utilization decreased from 230 to 139 DDDs per 1,000 patient-days between 2003 and 2019, whereas total antibiotic utilization increased from 1296 to 1550 DDDs per 1,000 patient-days. Infection Control interventions included empirically placing patients with diarrhea on precautions, intensified cleaning measures, formal antibiotic stewardship, introduction of a real-time PCR *C. difficile* test in June 2010, and a move to a facility with only single rooms at the RVH in April 2015.

Incidence of HA-CDI at the RVH and MGH and antibiotic utilization between 2003 and 2019

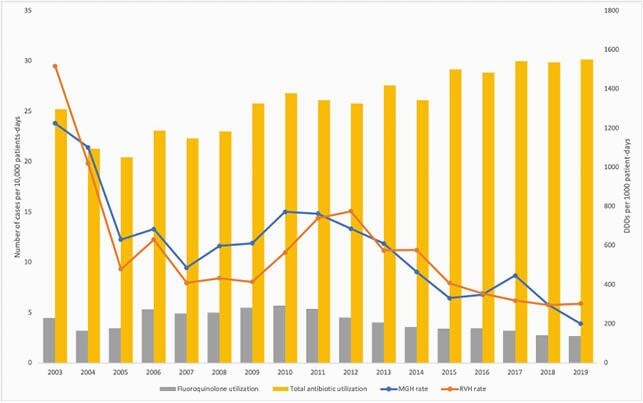

**Conclusion:**

An important change in HA-CDI epidemiology was observed in two Canadian tertiary care hospitals based in Montréal between 2003 and 2019. There was a significant decrease in incidence of HA-CDI and a genotype shift from a predominance of NAP1 strains to non-NAP1 strains. Utilization of fluoroquinolones, to which the NAP1 strain is resistant, concurrently decreased. Infection control interventions targeting isolation, diagnosis, disinfection, and antibiotic stewardship have contributed to the major observed reduction in HA-CDI incidence.

**Disclosures:**

**All Authors**: No reported disclosures

